# Tönnis Grade 1 dysplastic hips have improved patient-reported outcome scores when intraarticular pathology is treated during periacetabular osteotomy

**DOI:** 10.1093/jhps/hnab077

**Published:** 2021-10-28

**Authors:** Joseph A Panos, Claudia N Gutierrez, Cody C Wyles, Joshua S Bingham, Kristin C Mara, Robert T Trousdale, Rafael J Sierra

**Affiliations:** Mayo Clinic Alix School of Medicine, 200 1st St. SW, Rochester, MN 55905, USA; Mayo Clinic Alix School of Medicine, 200 1st St. SW, Rochester, MN 55905, USA; Department of Orthopedic Surgery, Mayo Clinic, 200 1st St. SW, Rochester, MN 55905, USA; Department of Orthopedic Surgery, Mayo Clinic, 5777 E Mayo Blvd, Phoenix, AZ 85054, USA; Division of Biomedical Statistics and Informatics, Mayo Clinic, 200 1st St. SW, Rochester, MN 55905, USA; Department of Orthopedic Surgery, Mayo Clinic, 200 1st St. SW, Rochester, MN 55905, USA; Department of Orthopedic Surgery, Mayo Clinic, 200 1st St. SW, Rochester, MN 55905, USA

## Abstract

It is unclear whether treatment of intraarticular pathology should be performed during periacetabular osteotomy (PAO) to improve outcomes. Therefore, we asked: (i) What are the clinical results of PAO in patients with and without intraarticular intervention? (ii) Is there a difference in reoperations with and without intraarticular intervention? and (iii) Is there a difference in clinical results and reoperations depending on preoperative Tönnis Grade if intraarticular intervention is performed? Prospective evaluation of 161 PAO in 146 patients was performed. The cohort was 84.5% female, mean age was 26.7 ± 7.9 years and mean follow-up was 2.4 years; 112 hips had Grade 0 changes and 49 hips had Grade 1 changes. Patients were classified into three groups based on treatments during PAO: major (labral repair, femoral head–neck osteochondroplasty), minor (labral debridement, femoral/acetabular chondroplasty) or no intervention. A subset of eight patient-reported outcome measures (PROMs) was analyzed to determine whether the minimal clinically important difference (MCID) was achieved. Major, minor and no intervention groups exceeded the MCID in 5, 8 and 8, of 8 PROMs (*P* ≥ 0.20), respectively; intraarticular interventions did not influence reoperation-free survival (*P* ≥ 0.35). By Tönnis Grade, PROMs exceeding MCID decreased in Grade 1 versus 0 receiving no intervention (*P* < 0.001) but did not decrease for either intervention (*P* ≥ 0.14); intraarticular interventions did not influence reoperation-free survival (*P* ≥ 0.38). Overall, intraarticular intervention was associated with excellent PROMs and reoperation-free survival. Although Grade 1 patients had fewer PROM which achieved MCID, intraarticular interventions attenuated this decrease, suggesting a therapeutic advantage of intraarticular procedures for more advanced pathology.

## INTRODUCTION

Developmental dysplasia of the hip (DDH) is associated with premature degenerative changes in the joint [1]. Nearly one in three dysplastic hips without radiographic evidence of arthritis (Tönnis Grade 0) will develop mild arthritic changes (Tönnis Grade 1) by 10 years, with one in four Tönnis Grade 0 patients being converted to total hip arthroplasty by 20 years [1]. Pelvic reorientation may be achieved in skeletally mature dysplastic patients with preserved articular cartilage via the Bernese periacetabular osteotomy (PAO) [2]. PAO has been shown to change the natural history of DDH by decreasing the risk of THA and degenerative changes in Tönnis Grade 0 and 1 hips [1, 3].

While recent 20- and 30-year follow-up reports have confirmed PAO to be an effective treatment for DDH [4, 5], a number of preoperative factors can influence the longevity and durability of the procedure. Namely, advanced preoperative radiographic Tönnis Grade has been associated with failure of PAO [6–8]. Degenerative changes to the hip conferring advanced Tönnis Grades are both preceded and potentiated by acetabular labral disease [9]. Most commonly occurring along the articular margin of the anterior portion of the acetabulum, labral tears can cause disruption of chondrolabral continuity, initiating the process of acetabular cartilage delamination [10]. Although the incidence of labral lesions has been reported to be up to 90% in patients with dysplasia [11], there is no consensus on the preferred management of labral pathology either contemporaneous or staged with PAO versus no treatment at all. As such, it is necessary to assess whether the addition of intraarticular interventions to PAO confers a clinical benefit or an elevated risk of reoperation over PAO alone. Accordingly, we asked: (i) What are the clinical results of PAO in patients with and without intraarticular intervention at the time of surgery? (ii) Is there a difference in reoperation rates after PAO with and without intraarticular intervention? and (iii) Is there a significant difference in clinical results and achievement of MCID and reoperations depending on the preoperative radiographic Tönnis Grade if intraarticular intervention is performed?

## METHODS

After obtaining Institutional Review Board (IRB No.: 17-001303) approval, we retrospectively reviewed all patients undergoing PAO at the Mayo Clinic in Rochester, Minnesota, between November 2009 and January 2016. All patients were treated by one of two senior hip preservation surgeons (R.T.T. or R.J.S.). Candidates for PAO had symptomatic DDH, defined by a lateral center-edge angle [12] <25°, acetabular index [13] >10° and anterior center-edge angle [14] <25°, with an age <50 years. We identified 171 patients (191 hips) patients who met these criteria. All patients undergoing surgical hip dislocation at the time of PAO were excluded (8 patients; 10 hips), all patients with non-DDH etiology of arthritic hip disease were excluded (13 patients, 16 hips) and all patients with Tönnis Grade ≥2 degenerative changes [15] were excluded (4 patients, 4 hips). Thus, the final cohort consisted of 146 patients (161 hips), with all patients having a diagnosis of symptomatic DDH.

As part of a prospectively collected hip preservation registry, 12 PROMs were recorded at the preoperative visit and each postoperative visit. In this cohort, the most recent clinical follow-up occurred at a mean of 2.4 years postoperatively (range: 0.8–5.7 years). PROMs included the University of California, Los Angeles (UCLA) activity score, Harris Hip Score, four subcomponents of the Hip Disability and Osteoarthritis Outcome Score (HOOS) [Pain, Activities of Daily Living (ADL), Sports and Recreation, Quality of Life], four subcomponents of the Western Ontario & McMaster Universities Questionnaire (WOMAC; Pain, Stiffness, Physical, Total) and two subcomponents of the SF-12 Health Survey (Physical and Mental). Each score has been used previously to assess the functional outcome of patients treated with PAO for symptomatic dysplasia [16–20]. For a subset of eight PROMs collected in this study, the preoperative to postoperative change was compared to the established minimal clinically important difference (MCID) reported in the literature [21].

Among the 146 patients (161 hips), 98 patients (105 hips) had preoperative Tönnis Grade 0 changes and 34 patients (42 hips) had Tönnis Grade 1 changes. Seven patients (14 hips) underwent staged bilateral PAO with side-to-side differences in Tönnis Grade. The cohort was 84.5% female, the mean age at the time of surgery was 26.7 ± 7.9 years (range: 12.7–47.7 years) and mean body mass index (BMI) was 25.5 ± 4.6 kg/m^2^ (range: 12.0–39.4 kg/m^2^) (Table I). During the study period, there was no defined indication for joint assessment. As per surgeon preference, either an arthrotomy or arthroscopy at the time of PAO was used to evaluate the joint. Labral repair and femoral head–neck osteochondroplasty were performed based on preoperative imaging identifying a tear or morphologic abnormality, respectively, in combination with patient symptomatology and functional goals following surgery. A femoral head–neck junction osteochondroplasty, for example, was added at the time of surgery to improve range of motion before impingement after correction. On this basis, each hip was subsequently re-classified by the extent of the intraarticular intervention performed at the time of PAO into either major (labral repair, femoral head–neck osteochondroplasty), minor (labral debridement, femoral/acetabular chondroplasty) or no intervention groups. By intraarticular intervention, Tönnis Grade 0 and Grade 1 groups did not differ by age, gender, BMI or the incidence of prior surgery to the affected hip (*P* ≥ 0.13) (Table I).

**Table I.** Patient characteristics by Tönnis Grade and intraarticular intervention

	*Tönnis Grade 0*				*Tönnis Grade 1*				
	*Major (n = 25)*	*Minor (n = 25)*	*No intervention (n = 62)*	*Adjusted P-value* *	*Major (n = 24)*	*Minor (n = 9)*	*No intervention (n = 16)*	*Adjusted P-value* *	*Total (n = 161)*
Age at surgery (years)				0.23				0.15	
Mean (SD)	26.8 (7.8)	26.5 (6.4)	24.1 (7.3)		30.9 (6.7)	34.1 (5.4)	26.6 (10.4)		26.7 (7.9)
Median	25.3	24.5	22.8		31.0	34.0	24.5		25.3
Q1, Q3	22.0, 33.4	20.8, 32.0	17.9, 29.6		27.2, 35.2	32.8, 37.5	17.7, 35.0		20.1, 32.6
Range	(15.4–42.3)	(17.9–39.1)	(14.0–43.3)		(19.2–46.1)	(23.5–41.7)	(12.7–47.7)		(12.7–47.7)
Gender				0.54				0.13	
F	20 (80.0%)	23 (92.0%)	56 (90.3%)		16 (66.7%)	8 (88.9%)	13 (81.3%)		136 (84.5%)
M	5 (20.0%)	2 (8.0%)	6 (9.7%)		8 (33.3%)	1 (11.1%)	3 (18.8%)		25 (15.5%)
BMI (lbs/in):				0.23				0.56	
Mean (SD)	26.2 (4.1)	24.4 (5.0)	24.4 (4.3)		28.2 (4.6)	26.2 (4.2)	26.0 (4.9)		25.5 (4.6)
Median	25.5	24.4	24.1		27.1	25.8	26.3		25.2
Q1, Q3	22.9, 29.1	20.9, 26.9	21.7, 26.7		25.0, 31.0	24.4, 27.9	22.7, 28.7		22.1, 27.9
Range	(20.3–34.7)	(17.6–39.4)	(12.0–35.4)		(20.8–37.7)	(20.4–34.7)	(18.5–38.6)		(12.0–39.4)
Side				0.32				0.27	
Left	12 (48.0%)	9 (36.0%)	20 (32.3%)		14 (58.3%)	4 (44.4%)	6 (37.5%)		65 (40.4%)
Right	13 (52.0%)	16 (64.0%)	42 (67.7%)		10 (41.7%)	5 (55.6%)	10 (62.5%)		96 (59.6%)
Any prior surgery?				0.58				0.82	
No	23 (92.0%)	22 (88.0%)	56 (90.3%)		19 (79.2%)	8 (88.9%)	11 (68.8%)		139 (86.3%)
Yes	2 (8.0%)	3 (12.0%)	6 (9.7%)		5 (20.8%)	1 (11.1%)	5 (31.3%)		22 (13.7%)
Method of joint interrogation									
Arthoscopy	4 (16.0%)	13 (52.0%)	2 (3.2%)		15 (62.5%)	3 (33.3%)	0 (0.0%)		37 (23.0%)
Arthrotomy	21 (84.0%)	12 (48.0%)	43 (69.4%)		9 (37.5%)	6 (66.7%)	7 (43.8%)		98 (60.9%)
None	0 (0.0%)	0 (0.0%)	17 (27.4%)		0 (0.0%)	0 (0.0%)	9 (56.2%)		26 (16.1%)
AP pelvic tilt (mm)				0.37				0.26	
Mean (SD)	47.3 (21.2)	54.4 (21.4)	54.3 (22.8)		45.1 (24.5)	42.0 (24.7)	36.7 (18.0)		49.3 (22.8)
Median	48.0	56.0	57.0		48.0	53.0	40.0		51.8
Q1, Q3	34.0, 66.0	43.5, 74.5	42.7, 70.0		24.0, 67.0	18.0, 60.0	22.0, 52.0		35.5, 65.2
Range	(0.0–79.0)	(8.0–89.7)	(4.3–106.0)		(2.5–87.0)	(4.0–71.0)	(4.5–60.0)		(0.0–106.0)
AP pelvic rotation (mm)				0.77				0.52	
Mean (SD)	4.6 (9.1)	2.7 (3.3)	4.0 (6.6)		1.6 (3.0)	3.6 (4.2)	3.3 (3.9)		3.4 (5.9)
Median	1.0	0.0	4.0		0.0	2.0	2.0		0.0
Q1, Q3	0.0, 5.0	0.0, 5.0	0.0, 5.0		0.0, 2.0	0.0, 5.0	0.0, 7.0		0.0, 5.0
Range	(0.0–41.2)	(0.0–10.0)	(0.0–43.1)		(0.0–10.0)	(0.0–10.0)	(0.0–10.0)		(0.0–43.1)
Lateral center-edge angle (degrees)				0.58				0.27	
Mean (SD)	17.0 (7.5)	16.5 (5.7)	15.2 (6.5)		15.7 (7.4)	14.5 (7.5)	12.8 (9.9)		15.5 (7.1)
Median	17.0	19.0	15.0		16.5	18.0	15.0		16.0
Q1, Q3	11.0, 20.0	13.0, 20.0	10.0, 20.0		12.5, 21.0	10.0, 19.0	9.5, 19.3		11.0, 20.0
Range	(4.0–37.4)	(2.1–24.9)	(0.0–29.0)		(−3.0–25.0)	(0.0–25.0)	(−18.0–23.0)		(−18.0–37.4)
Acetabular inclination (degrees)				0.21				0.046	
Mean (SD)	15.9 (6.5)	16.3 (8.1)	18.8 (7.2)		14.8 (6.2)^a^	21.7 (9.7)^a^	20.6 (8.8)^a^		17.7 (7.6)
Median	15.0	14.0	17.0		12.6	24.0	21.5		15.0
Q1, Q3	11.0, 21.8	10.0, 21.0	13.0, 24.0		11.0, 17.0	15.0, 26.7	13.9, 23.5		12.0, 23.0
Range	(2.3–25.0)	(6.1–33.0)	(6.5–40.0)		(5.0–29.0)	(8.0–40.0)	(8.6–40.0)		(2.3–40.0)

* All *P*-values comparing intraarticular intervention have been adjusted for surgical group.

a Post hoc pairwise comparisons, connecting letters report: groups that share the same letter do not differ statistically.

The number and the nature of postoperative reoperations and complications were confirmed in the medical record. Isolated hardware removal stemming from index PAO was not considered in the assessment of the incidence of reoperation. Subsequent hip arthroscopy after index PAO was performed on patients with persistent pain or dysfunction that failed non-operative treatment independent of their original procedure. The modified Dindo–Clavien Classification scheme was used to grade all recorded complications following PAO [22]. Briefly, Grade I complications required no treatment or alteration to the postoperative protocol, Grade II complications required pharmacological or additional outpatient follow-up, Grade III complications necessitated surgical intervention and Grade IV complications were untreatable and caused permanent disability or death. The reliability of this classification system has been previously demonstrated to grade complications following hip preservation surgery [23].

The data are presented as counts and percentages for categorical variables or means and standard deviations for continuous variables. Comparisons of baseline characteristics and PROM scores (preoperative, postoperative, the change from preoperative to postoperative and the difference between the preoperative to postoperative change and MCID) were made using generalized estimating equations to account for the fact that a patient may have more than one hip included in the analysis. Where appropriate, post hoc pairwise comparisons were conducted using the generalized estimating equations with *P*-values adjusted for multiple comparisons using the Benjamini–Hochberg false discovery rate method [24]. The PROM exceeding the MCID was compared between Tönnis Grades 0 and 1 using the Fisher’s exact test. Cox proportional hazards regression with a robust variance estimator was used to assess the incidence of reoperations following PAO. All analyses were performed using SAS version 9.4 (SAS Institute Inc., Cary, NC) and R version 3.4.2 (R Core Team, Vienna, Austria).

## RESULTS

For the entire cohort, patients receiving major, minor or no intraarticular intervention at index PAO exceeded the MCID in 5, 8 and 8, of 8 PROMs (*P* ≥ 0.20), respectively (Table II).

**Table II.** Preoperative and postoperative patient-reported outcome measures, by intraarticular intervention performed

	*Major (n = 49)*	*Minor (n = 34)*	*No intervention (n = 78)*	*Total (n = 161)*	*Adjusted P-value* *
UCLA score					
Preoperative	6.1 (2.3)	6.1 (2.5)	7.1 (2.6)	6.6 (2.5)	0.056
Postoperative	7.0 (2.2)	7.4 (2.2)	8.2 (1.8)	7.6 (2.1)	0.014
Change (post–pre)	1.1 (2.9)	1.4 (2.3)	0.9 (2.7)	1.1 (2.7)	0.75
*P*-value	0.024	0.005	0.020	<0.001	
Harris Hip Score					
Preoperative	65.1 (14.0)	59.2 (14.3)	63.6 (14.7)	63.1 (14.5)	0.21
Postoperative	86.5 (15.1)	84.3 (16.5)	88.8 (11.9)	87.0 (14.1)	0.40
Change (post–pre)	23.2 (15.3)	24.8 (16.5)	23.3 (16.4)	23.6 (16.0)	0.91
*P*-value	<0.001	<0.001	<0.001	<0.001	
HOOS Total Pain					
Preoperative	56.5 (16.8)	48.8 (17.4)	58.1 (19.0)	55.7 (18.3)	0.061
Postoperative	85.2 (18.2)	82.9 (17.2)	87.4 (14.6)	85.7 (16.3)	0.49
Change (post–pre)	27.8 (19.1)	32.8 (18.6)	28.4 (21.7)	29.2 (20.2)	0.54
*P*-value	<0.001	<0.001	<0.001	<0.001	
MCID (10.3) *P*-value	<0.001	<0.001	<0.001	<0.001	
HOOS Total ADL					
Preoperative	70.4 (20.2)	63.4 (19.9)	73.4 (19.8)	70.5 (20.2)	0.10
Postoperative	90.2 (15.3)	88.6 (15.8)	93.8 (9.0)	91.5 (13.0)	0.19
Change (post–pre)	18.0 (21.1)	22.7 (18.0)	17.7 (19.5)	18.9 (19.6)	0.51
*P*-value	<0.001	<0.001	<0.001	<0.001	
MCID (10.8) *P*-value	0.054	0.002	0.011	<0.001	
HOOS Total S&R					
Preoperative	40.2 (22.9)	34.4 (22.4)	46.4 (21.4)	42.0 (22.4)	0.039
Postoperative	78.2 (22.3)	74.8 (23.7)	81.4 (18.0)	78.9 (20.7)	0.42
Change (post–pre)	39.4 (30.5)	36.4 (26.6)	32.1 (25.1)	35.4 (27.2)	0.44
*P*-value	<0.001	<0.001	<0.001	<0.001	
MCID (12.6) *P*-value	<0.001	<0.001	<0.001	<0.001	
HOOS Total QOL					
Preoperative	29.3 (17.4)	26.2 (17.9)	34.2 (17.3)	31.0 (17.7)	0.090
Postoperative	68.9 (23.9)	67.2 (19.4)	74.8 (18.3)	71.3 (20.5)	0.19
Change (post–pre)	39.6 (26.8)	42.1 (21.3)	38.0 (24.6)	39.4 (24.4)	0.75
*P*-value	<0.001	<0.001	<0.001	<0.001	
MCID (11.2) *P*-value	<0.001	<0.001	<0.001	<0.001	
WOMAC Total Pain					
Preoperative	62.4 (17.9)	53.8 (18.4)	63.5 (19.3)	61.2 (18.9)	0.055
Postoperative	86.9 (17.7)	86.9 (15.7)	91.1 (12.7)	88.9 (15.0)	0.31
Change (post–pre)	23.7 (20.8)	32.3 (17.3)	25.5 (21.8)	26.5 (20.6)	0.17
*P*-value	<0.001	<0.001	<0.001	<0.001	
MCID (10.8) *P*-value	<0.001	<0.001	<0.001	<0.001	
WOMAC Total Stiffness					
Preoperative	58.5 (23.6)	49.6 (21.6)	59.8 (25.0)	57.3 (24.1)	0.10
Postoperative	76.3 (23.4)	78.6 (22.0)	83.5 (19.1)	80.2 (21.2)	0.26
Change (post–pre)	18.1 (31.8)	28.4 (19.5)	20.2 (26.3)	21.3 (26.9)	0.18
*P*-value	0.002	<0.001	<0.001	<0.001	
MCID (12.9) *P*-value	0.33	<0.001	0.038	<0.001	
WOMAC Total Physical					
Preoperative	70.4 (20.2)	63.4 (19.9)	73.4 (19.8)	70.5 (20.2)	0.10
Postoperative	90.3 (15.4)	88.9 (15.3)	93.9 (9.1)	91.7 (12.9)	0.20
Change (post–pre)	18.0 (21.4)	23.0 (18.0)	17.8 (19.4)	19.0 (19.7)	0.47
*P*-value	<0.001	<0.001	<0.001	<0.001	
MCID (10.8) *P*-value	0.061	0.001	0.009	<0.001	
WOMAC Total					
Preoperative	68.1 (18.8)	60.0 (18.9)	70.3 (19.5)	67.6 (19.4)	0.072
Postoperative	88.6 (15.8)	87.3 (15.6)	92.7 (9.5)	90.2 (13.3)	0.16
Change (post–pre)	18.9 (20.3)	25.5 (17.2)	19.9 (19.4)	20.8 (19.2)	0.37
*P*-value	<0.001	<0.001	<0.001	<0.001	
^b^MCID (10.4) *P*-value	0.018	<0.001	<0.001	<0.001	
SF12 Physical					
Preoperative	39.2 (10.2)	36.9 (11.0)	41.1 (10.2)	39.6 (10.4)	0.18
Postoperative	51.2 (8.9)	47.7 (11.7)	53.3 (7.1)	51.4 (9.1)	0.065
Change (post–pre)	12.6 (11.1)	11.0 (12.7)	11.7 (10.8)	11.8 (11.3)	0.86
*P*-value	<0.001	<0.001	<0.001	<0.001	
SF12 Mental					
Preoperative	52.5 (10.3)	54.8 (9.5)	53.1 (11.2)	53.3 (10.6)	0.56
Postoperative	49.1 (13.3)	57.2 (7.0)	52.5 (9.5)	52.5 (10.8)	0.008
Change (post–pre)	−3.1 (12.9)	2.3 (11.1)	−1.0 (10.6)	−0.9 (11.6)	0.21
*P*-value	0.16	0.27	0.47	0.43	

* All *P*-values comparing intraarticular intervention have been adjusted for surgical group.

b Minimal clinically important difference [21].

The cumulative incidence of reoperation-free survival (excluding hardware removal) at 1 year was 100% [95% confidence interval (95% CI) = 100–100%] and at 2 years was 94.3% (95% CI = 87.3–100%) for patients receiving major intraarticular interventions. For patients receiving minor interventions, reoperation-free survival was 93.9% (95% CI = 84.8–100%) at 1 and 2 years. Reoperation-free survival was 95.5% (95% CI = 90.7–100%) at 1 year and 89.8% (95% CI = 82.3%-98.0%) for patients receiving no intraarticular intervention. There was no difference in the risk of reoperation between groups [major versus no intervention: hazard ratio (HR) = 0.54; 95% CI = 0.14–2.03, *P* = 0.36; minor versus no intervention HR = 1.71; 95% CI = 0.55–5.29, *P* = 0.35] (Table III).

**Table III.** Reoperation-free survival by intraarticular intervention

*Reoperation-free survival (cumulative incidence)*				
*Time (years)*	*Major (95% CI)*	*Minor (95% CI)*	*No intervention (95% CI)*	*Overall (95% CI)*
1	100% (100–100)	93.3% (84.8–100)	95.5% (90.7–100)	96.5% (93.6–99.6)
2	94.3% (87–100)	93.3% (84.8–100)	89.8% (82.3–98)	92.0% (87.3–96.9)
*Cox proportional hazards ratio*				
*Major (95% CI)*	*Major P-value*	*Minor (95% CI)*	*Minor P-value*	*No intervention*
0.54 (0.14–2.03)	0.36	1.71 (0.55–5.29)	0.35	Reference

When the cohort was divided by preoperative Tonnis Grade, Grade 0 patients undergoing major, minor or no intervention exceeded the MCID in 7, 8 and 8 of 8 PROMs, respectively. In Tönnis Grade 1 patients, major, minor and no intervention exceeded the MCID in 4, 7 and 1 of 8 PROMs, respectively (Table IV). The proportion of PROM exceeding MCID significantly decreased between Tönnis Grade 0 and Grade 1 patients receiving no intervention (*P* < 0.001) but did not decrease for patients receiving major or minor interventions (*P* ≥ 0.14). The cumulative incidence of reoperation-free survival (excluding hardware removal) at 1 year was 96.6% (95% CI = 93.6–100%) and at 2 years was 94.5% (95% CI = 89.9–99.3%) for Tönnis Grade 0 patients. For Tönnis Grade 1 patients, reoperation-free survival was 95.7% (95% CI = 89.9–100%) at 1 year and 86.1% (95% CI = 75.3–98.5%) at 2 years. Interestingly, reoperation-free survival did not differ between patients receiving a major or minor intervention relative to no intervention for Tönnis Grade 0 or Grade 1 patients (*P* ≥ 0.38) (Table V).

**Table IV.** Preoperative and postoperative patient-reported outcome measures, by Tönnis Grade intraarticular intervention performed

	*Tönnis Grade 0*				*Tönnis Grade 1*			
	*Major (n = 25)*	*Minor (n = 25)*	*No intervention (n = 62)*	*Adjusted P-value* *	*Major (n = 24)*	*Minor (n = 9)*	*No intervention (n = 16)*	*Adjusted P-value* *
UCLA score								
Preoperative	5.6 (2.2)	5.9 (2.5)	7.1 (2.6)	0.014	6.8 (2.4)	6.5 (2.6)	7.2 (2.6)	0.75
Postoperative	7.4 (2.2)	7.6 (2.1)	8.3 (1.6)	0.072	6.5 (2.2)	7.0 (2.5)	7.9 (2.2)	0.58
Change (post–pre)	1.9 (3.2)	1.3 (2.2)	1.1 (2.9)	0.45	0.2 (2.5)	1.4 (2.7)	0.5 (2.1)	0.69
*P*-value	0.017	0.010	0.025		0.75	0.31	0.45	
Harris Hip Score								
Preoperative	64.3 (16.9)	60.2 (14.0)	62.9 (13.4)	0.52	65.9 (10.1)	56.2 (15.9)	67.4 (20.6)	0.21
Postoperative	90.6 (10.3)	86.8 (14.4)	89.7 (10.5)	0.37	82.9 (17.9)	77.8 (20.8)	86.0 (15.6)	0.67
Change (post–pre)	25.7 (17.7)	26.4 (16.7)	25.2 (15.3)	0.96	20.6 (12.5)	20.1 (16.3)	15.4 (19.3)	0.40
*P*-value	<0.001	<0.001	<0.001		<0.001	0.031	0.055	
HOOS Total Pain								
Preoperative	57.1 (17.9)	50.7 (18.3)	57.6 (18.8)	0.14	55.9 (15.9)	42.9 (13.2)	60.6 (20.8)	0.10
Postoperative	89.2 (12.2)	84.3 (17.1)	88.4 (13.9)	0.45	81.3 (22.3)	79.4 (18.4)	84.1 (16.8)	0.82
Change (post–pre)	28.1 (19.3)	33.3 (20.1)	30.4 (21.5)	0.33	27.5 (19.4)	31.3 (14.3)	22.1 (22.2)	0.49
*P*-value	<0.001	<0.001	<0.001		<0.001	0.031	0.005	
^c^MCID (10.3) *P*-value	0.001	<0.001	<0.001		0.002	0.016	0.093	
HOOS Total ADL								
Preoperative	66.2 (19.8)	64.4 (21.3)	72.0 (20.3)	0.11	75.9 (20.0)	61.0 (16.9)	80.4 (16.4)	0.049
Postoperative	94.8 (6.0)	88.7 (16.7)	95.3 (5.8)	0.21	85.3 (20.2)	88.4 (14.4)	89.4 (14.7)	0.87
Change (post–pre)	24.3 (16.7)	22.1 (19.7)	20.7 (20.3)	0.38	9.6 (23.9)^a,b^	23.9 (14.3)^b^	7.4 (12.4)^a^	0.029
*P*-value	<0.001	<0.001	<0.001		0.14	0.016	0.078	
^c^MCID (10.8) *P*-value	0.002	0.031	0.003		0.85	0.052	0.36	
HOOS Total S&R								
Preoperative	37.8 (23.5)	36.1 (23.1)	44.9 (21.5)	0.054	42.9 (22.4)	28.6 (20.7)	53.4 (20.5)	0.075
Postoperative	82.2 (18.9)	75.7 (25.8)	81.8 (17.0)	0.43	74.0 (25.3)	72.7 (19.5)	79.7 (22.0)	0.79
Change (post–pre)	44.1 (29.9)	34.9 (28.8)	34.8 (24.1)	0.24	34.2 (31.2)	40.6 (20.8)	20.6 (27.2)	0.15
*P*-value	<0.001	<0.001	<0.001		<0.001	0.031	0.063	
^c^MCID (12.6) *P*-value	<0.001	0.006	<0.001		0.011	0.022	0.38	
HOOS Total QOL								
Preoperative	27.8 (17.7)	25.0 (19.2)	34.2 (18.2)	0.065	31.3 (17.3)	29.7 (14.1)	33.9 (13.4)	0.49
Postoperative	73.7 (19.0)	67.3 (19.7)	75.3 (17.2)	0.21	64.1 (27.6)	67.0 (20.3)	73.2 (22.4)	0.92
Change (post–pre)	45.4 (25.9)	43.8 (21.6)	38.9 (24.4)	0.38	32.8 (27.1)	37.5 (21.7)	35.1 (26.2)	0.92
*P*-value	<0.001	<0.001	<0.001		<0.001	0.016	<0.001	
^c^MCID (11.2) *P*-value	<0.001	<0.001	<0.001		0.006	0.018	0.006	
WOMAC Total Pain								
Preoperative	62.2 (19.3)	55.9 (19.6)	62.3 (19.5)	0.30	62.7 (16.5)^a^	47.1 (12.5)^b^	68.6 (18.1)^a^	0.046
Postoperative	90.0 (12.1)	86.9 (17.0)	91.8 (12.3)	0.52	83.9 (21.6)	86.9 (12.5)	88.9 (14.0)	0.94
Change (post–pre)	24.2 (20.7)	30.8 (18.9)	28.0 (22.4)	0.44	23.2 (21.6)	37.5 (9.4)	17.3 (18.1)	0.064
*P*-value	<0.001	<0.001	<0.001		<0.001	0.031	0.004	
^c^MCID (10.8) *P*-value	0.014	<0.001	<0.001		0.031	0.031	0.22	
WOMAC Total Stiffness								
Preoperative	55.0 (20.4)	48.4 (22.2)	59.2 (25.4)	0.067	62.5 (26.7)	53.1 (20.9)	62.5 (24.0)	0.52
Postoperative	81.9 (19.8)	76.3 (22.2)	84.2 (19.7)	0.44	71.3 (25.7)	84.4 (21.9)	81.3 (17.5)	0.49
Change (post–pre)	23.6 (30.3)	28.3 (21.6)	21.6 (26.3)	0.19	12.5 (33.2)	28.6 (13.9)	15.4 (26.6)	0.26
*P*-value	0.007	<0.001	<0.001		0.15	0.016	0.082	
^c^MCID (12.9) *P*-value	0.15	0.006	0.034		0.96	0.024	0.74	
WOMAC Total Physical								
Preoperative	66.2 (19.8)	64.4 (21.3)	72.0 (20.3)	0.11	75.9 (20.0)^a^	61.0 (16.9)^a^	80.4 (16.4)^b^	0.049
Postoperative	94.9 (6.2)	88.7 (16.7)	95.4 (5.9)	0.20	86.1 (20.0)	89.5 (12.1)	89.4 (14.7)	0.82
Change (post–pre)	24.5 (17.1)	22.1 (19.7)	20.8 (20.2)	0.39	9.6 (23.9)^a^	25.2 (14.3)^a^	7.4 (12.4)^b^	0.026
*P*-value	<0.001	<0.001	<0.001		0.14	0.016	0.078	
^c^MCID (10.8) *P*-value	0.003	0.031	0.003		0.85	0.037	0.36	
WOMAC Total								
Preoperative	64.5 (18.6)	61.6 (20.2)	68.7 (19.9)	0.15	72.8 (18.5)^a^	55.4 (14.6)^b^	77.6 (15.8)^a^	0.034
Postoperative	92.9 (7.7)	86.8 (17.0)	94.0 (7.0)	0.18	84.4 (20.5)	88.5 (12.2)	88.6 (14.4)	0.81
Change (post–pre)	24.4 (16.9)	24.2 (18.7)	23.2 (20.0)	0.57	11.5 (22.7)^a^	28.8 (13.2)^b^	9.3 (12.7)^a^	0.025
*P*-value	<0.001	<0.001	<0.001		0.11	0.031	0.023	
^c^MCID (10.4) *P*-value	0.002	<0.001	<0.001		0.86	0.031	0.77	
SF12 Physical								
Preoperative	38.0 (9.5)	37.9 (10.9)	41.4 (9.6)	0.11	40.5 (11.1)	34.0 (11.3)	39.5 (12.6)	0.18
Postoperative	54.6 (6.9)	48.2 (11.6)	54.6 (6.1)	0.14	48.3 (9.6)	46.7 (12.6)	49.5 (8.7)	0.74
Change (post–pre)	15.9 (7.1)	10.0 (11.4)	12.5 (11.3)	0.036	9.3 (13.5)	13.8 (16.8)	9.1 (9.3)	0.77
*P*-value	<0.001	<0.001	<0.001		0.008	0.078	0.002	
SF12 Mental								
Preoperative	51.8 (9.2)	53.2 (9.9)	52.4 (11.5)	0.86	53.3 (11.6)	59.8 (6.1)	55.9 (10.1)	0.29
Postoperative	49.2 (14.4)	57.6 (6.3)	52.3 (10.6)	0.18	48.9 (12.6)	56.0 (8.9)	53.1 (5.7)	0.45
Change (post–pre)	−2.2 (14.1)	4.4 (10.6)	−0.6 (11.3)	0.39	−4.1 (11.9)	−4.0 (11.1)	−2.5 (7.9)	0.97
*P*-value	0.77	0.11	0.78		0.086	0.58	0.31	

* All *P*-values comparing intraarticular intervention have been adjusted for surgical group.

a,b Post hoc pairwise comparisons, connecting letters report: groups that share the same letter do not differ statistically.

c Minimal clinically important difference [21].

**Table V.** Reoperation-free survival by intraarticular intervention and Tönnis Grade

*Tönnis Grade 0*				
*Reoperation-free survival (cumulative incidence)*				
*Time (years)*	*Major (95% CI)*	*Minor (95% CI)*	*No intervention (95% CI)*	*Overall (95% CI)*
1	100% (100–100)	95.5% (87.1–100)	96.2% (91.1–100)	96.6% (93.6–100)
2	95% (85.9–100)	95.5% (87.1–100)	93.8% (87.2–100)	94.5% (89.9–99.3)
*Cox proportional hazards ratio*				
*Major (95% CI)*	*Major P-value*	*Minor (95% CI)*	*Minor P-value*	*No intervention*
0.45 (0.05–4.02)	0.47	1.97 (0.44–8.86)	0.38	Reference
*Tönnis Grade 1*				
*Reoperation-free survival (cumulative incidence)*				
*Time (years)*	*Major (95% CI)*	*Minor (95% CI)*	*No intervention (95% CI)*	*Overall (95% CI)*
1	100% (100–100)	87.5% (67.3–100)	93.3% (81.5–100)	95.7% (89.9–100)
2	92.9% (80.3–100)	87.5% (67.3–100)	76.4% (56–100)	86.1% (75.3–98.5)
*Cox proportional hazards ratio*				
*Major (95% CI)*	*Major P-value*	*Minor (95% CI)*	*Minor P-value*	*No intervention*
0.47 (0.08–2.63)	0.39	1.25 (0.22–7.06)	0.80	Reference

All reoperations and complications following index PAO are summarized in Tables VI and VII. Notably, eight patients (eight hips; 22.9%) underwent subsequent hip arthroscopy at an average of 2.7 years (range: 0.8–4.6 years) following index PAO: three patients (three hips) with no previous hip arthroscopy, and five patients (five hips) had previously undergone hip arthroscopy or arthrotomy. One patient (one hip) underwent reoperation with a major intraarticular intervention (femoral head–neck osteochondroplasty) 2.3 years after initially receiving PAO alone. There were no Grade IV complications. The rate of major (Grade III or IV) complications was 2.4%.

**Table VI.** Reoperations following index PAO

*Patients (hips; %)*	*Reoperation*	*Time from index PAO (range)* *^a^* *Time from primary reoperation (range)*
23 (25; 15.5%); ^a^2 (2; 1.2%)	Hardware removal; ^a^Wound I&D	1.3 years (0.4–5.3 years); ^a^8 weeks (3–13 weeks)
2 (2; 1.2%); ^a^1 (1; 0.6%)	Wound I&D; ^a^Drain placement	7 weeks (6.5–7.5 weeks); ^a^3 weeks ()
8 (8; 5.0%); ^a^1 (1; 0.6%)	Hip arthroscopy; ^a^Wound I&D	2.7 years (0.8–4.6 years); ^a^3 weeks ()
1 (1; 0.6%)	Hip arthrotomy	2.3 years ()
1 (1; 0.6%)	Saphenous nerve biopsy	6 weeks ()
1 (1; 0.6%)	Correcting PAO	3.0 years ()

a Indicates a second procedure stemming from an initial reoperation.

**Table VII.** Complications according to Dindo–Clavien grading system (Grades I–IV)

*Complication*	*Major intervention (hips; %)*	*Minor intervention (hips; %)*	*No intervention (hips; %)*	*Overall (hips; %)*
Grade I				
LFCN dysesthesia	4 (4; 2.5)	4 (4; 2.5)	4 (4; 2.5)	12 (12; 7.5)
Grade II				
DVT	1 (1; 0.6)	0 (0; 0.0)	1 (1; 0.6)	2 (2; 1.2)
Femoral n. palsy	1 (1; 0.6)	1 (1; 0.6)	0 (0; 0.0)	2 (1; 1.2)
Lumbar plexopathy, pharmalogical intervention	0 (0; 0.0)	0 (0; 0.0)	1 (1; 0.6)	1 (1; 0.6)
Stress fracture, inferior pubic ramus	0 (0; 0.0)	0 (0; 0.0)	1 (1; 0.6)	1 (1; 0.6)
Grade III				
Lumbar plexopathy, surgical intervention (saphenous n. biopsy)	0 (0; 0.0)	0 (0; 0.0)	1 (1; 0.6)	1 (1; 0.6)
HO, requiring surgical excision	1 (1; 0.6)	0 (0; 0.0)	0 (0; 0.0)	1 (1; 0.6)
Deep wound infection	0 (0; 0.0)	1 (1; 0.6)	1 (1; 0.6)	2 (2; 1.2)

## DISCUSSION

This study evaluated whether Tönnis Grade or adjunctive intraarticular interventions performed during PAO influenced PROM, reoperation-free survival or the incidence of reoperation in patients with DDH. For patients without evidence of arthritis (Tönnis Grade 0), both PAO alone and PAO with intraarticular treatment of identified pathology produced reliable and clinically meaningful improvements in PROM. A significant decrease in the proportion of PROM achieving MCID occurred between Tönnis Grade 0 and Grade 1 patients who did not receive intraarticular treatment. In contrast, those Tönnis Grade 1 patients that underwent intraarticular interventions did not experience a significant decrease in PROM which achieved MCID. Despite these clinically significant differences, there was no difference in reoperation rates.

A number of limitations are associated with this study. First, use of adjuvant arthrotomy or arthroscopy to identify intracapsular pathology was performed non-systematically at the discretion of the treating surgeon. In a number of cases, patients classified as receiving no intraarticular intervention underwent concomitant arthroscopy or arthrotomy; however, no procedure was performed within the joint. This group served as an imperfect baseline to which intraarticular intervention could be compared. Matching patients with similar intraarticular pathology, identified via intraoperative joint inspection, with and without intervention would more effectively delineate the role of intraarticular therapy on PROM following PAO. Second, the relatively short-term follow-up period of this study precludes the ability to determine the effect of surgical technique or intraarticular intervention on the arthritic progression of the dysplastic hip after PAO. Specifically, by 5 years postoperatively, the majority of DDH patients with Tönnis Grade 0 or 1 morphology have not progressed to subsequent Tönnis Grades [3]. In this manner, long-term follow-up is necessary to determine the influence of intraarticular intervention simultaneous with PAO. Third, this study did not account for other factors that may influence PROM or natural history following PAO, namely the accuracy and degree of acetabular correction. Recent data have shown that patients with more severe baseline DDH experience greater improvements in PROM [25], and the natural history of the native hip is improved with the restoration of ‘normal’ radiographic parameters of the acetabular fragment [26].

Treatment of the labrum during PAO remains controversial. At 10-year follow-up of the initial Bern cohort, Siebenrock *et al*. identified a labral tear as a predictor of inferior outcomes following PAO [27]. Alternatively, Goronzy *et al*. reported a cohort of 86 patients (106 hips) undergoing either PAO alone versus PAO with arthrotomy or arthroscopy with a major intraarticular intervention (osteochondroplasty). The group receiving PAO with arthroscopy additionally underwent treatment of labral pathology or chondral lesions, as indicated. At mean follow-up of 63 months, no differences in PROM or conversion to THA were identified between the three groups [28]. Without distinguishing statistical versus clinical improvement, Goronzy did not recommend joint inspection at the time of PAO. Notably, the majority of hips with complete follow-up in this study (57 of 66; 86%) had preoperative Kellgren–Lawrence Grade 0 arthritic changes. At 4.5-year follow-up in 22 patients with a preoperative labral tear and no evidence of arthritis, Pitto *et al*. found that outcomes following PAO pertaining to pain were not influenced by intraarticular treatment of the labrum [29]. In this regard, the results of the present study for Tönnis Grade 0 patients, demonstrating clinically significant improvement in almost all PROMs by surgical technique or intraarticular intervention, are in agreement with Goronzy and Pitto. However, Tonnis Grade 1 patients may have a number of pain generators and more advanced labral or cartilage damage that may necessitate intraarticular inspection and treatment. In comparing those PROMs which achieved MCID between Tönnis Grade 0 and Grade 1 patients, a significant decrease in PROM achieving clinical improvement occurred in those patients with Tönnis Grade 1 arthritis and no intraarticular intervention. In this manner, intraarticular treatment in excess of the mechanical offloading achieved in PAO may be more consequential for patients with mild, Tönnis Grade 1 arthritic changes. However, longer follow-up in this cohort is necessary to monitor PROM and to delineate the effects of chondrolabral treatment at index PAO on the progression of arthritic changes to the joint. Defining and classifying the labral pathology or other intraarticular pain generators with greater granularity would contribute to the preoperative identification of those patients most likely to benefit from intraarticular treatment at the time of PAO.

Overall, excellent short-term PROMs may be achieved with intraarticular intervention performed at the time of PAO, without increasing the risk of reoperation versus no intervention. Clinically significant improvement in PROM was observed in Tönnis Grade 0 patients following PAO. In patients with Tönnis Grade 1 arthritic changes who did not receive an intraarticular intervention, the proportion of PROM which achieved MCID significantly decreased compared to Tönnis Grade 0 patients. Intraarticular treatment targeted at the labrum or cartilage attenuated the decrease in PROM which achieved MCID in Tönnis Grade 1 patients, suggesting a therapeutic advantage of such interventions for patients with more advanced pathology. Despite improvements in PROM scores in Tonnis Grade 1 hips, there was no difference in reoperation rates between the groups. That is, despite not achieving MCID in certain measures, patients did not seem to be symptomatic enough to warrant subsequent surgical intervention. Further follow-up is needed to determine whether patients will have worsening pain and function that may warrant subsequent procedures.

## Data Availability

All data are incorporated into the article and its online supplementary material.
